# Supplementary material for: Kondo effect and spin–orbit coupling in graphene quantum dots
Source: Nat Commun. 2021 Oct 14;12:6004. doi: 10.1038/s41467-021-26149-3 (PMC8516925; doi:10.1038/s41467-021-26149-3)
Supplement: Supplementary file 1 — Supplementary Information [file 41467_2021_26149_MOESM1_ESM.pdf]

Supplementary Information: Kondo effect and spin-orbit coupling in graphene quantum dots

Annika Kurzmann,[1,a)] Yaakov Kleeorin,[2] Chuyao Tong,[1] Rebekka Garreis,[1] Angelika Knothe,[3] Marius Eich,[1] Christopher Mittag,[1] Carolin Gold,[1] F.K. de Vries,[1] Kenji Watanabe,[4] Takashi Taniguchi,[4] Vladimir Fal'ko,[3] Yigal Meir,[5] Thomas Ihn,[1,6] and Klaus Ensslin[1,6]

[1)] *Solid State Physics Laboratory, ETH Zürich, CH-8093 Zürich, Switzerland*

[2)] *Center for the Physics of Evolving Systems, Biochemistry and Molecular Biology, University of Chicago , Chicago, IL, 60637, USA*

[3)] *National Graphene Institute, University of Manchester, Manchester M13 9PL, United Kingdom*

[4)] *National Institute for Material Science, 1-1 Namiki, Tsukuba 305-0044, Japan*

[5)] *Department of Physics, Ben-Gurion University of the Negev, Beer-Sheva, 84105, Israel*

[6)] *Quantum Center, ETH Zurich, 8093 Zurich, Switzerland*

(Dated: 5 October 2021)

[a)] Electronic mail: annikak@phys.ethz.ch

SUPPLEMENTARY NOTE 1: NRG CALCULATION

We model the quantum dot in bilayer graphene system by a degenerate state with two quantum numbers $\sigma = \pm 1/2$ spin denoted $\uparrow, \downarrow$ and $v = \pm 1/2$ valley denoted k, k'

$$H_{dot} = \sum_{\sigma v} (\epsilon_d + \frac{1}{2}\Delta_{so} v\sigma) n_{\sigma v} + U \sum_{\{\sigma v\} \neq \{\sigma' v'\}} n_{\sigma v} n_{\sigma' v'} + J \vec{S}_k \cdot \vec{S}_{k'} \tag{1}$$

where $n_{\sigma v} = d^\dagger_{\sigma v} d_{\sigma v}$ is the occupation number operator of valley v with spin σ, Δ_{so} is the energy gap between the two spin-orbit split Kramer pairs (each pair characterized by a different value of the product $v\sigma$), U is the Coulomb repulsion between electrons in different states , and J is the magnetic interaction between electrons in the two valleys, that ensures a valley-singlet two-particle ground state, as seen experimentally. The spin vector for valley v is defined as $\vec{S}_v = \frac{1}{2} \sum_{\sigma, \sigma'} d^\dagger_{v\sigma} \vec{\sigma}_{\sigma\sigma'} d_{v\sigma'}$, where $\vec{\sigma}$ are the Pauli matrices. The system is connected to leads on both sides $s = L/R$ by the tunneling Hamiltonian

$$H_{tun} = \sum_{s,p} \sum_{\sigma v} V_s c^\dagger_{sp\sigma v} d_{\sigma v} + h.c \tag{2}$$

where $c^\dagger_{sp\sigma v}$ is the creation operator of an electron on side s with momentum p, spin σ and valley quantum number v, and it is assumed that the tunneling process conserves spin and valley degrees of freedom. The electrons in the leads are described by the non-interacting Hamiltonian

$$H_{res} = \sum_{s,p} \sum_{\sigma, v} \epsilon_{sp} c^\dagger_{sp\sigma v} c_{sp\sigma v} \tag{3}$$

We assume equal tunneling to the left and right reservoirs, $\Gamma_{L/R} = \Gamma = \pi\rho V_{L/R}$, and equal and constant density of states ρ in the two leads, whose bandwidth is defined as the unit of energy in the calculations.

The numerical calculations required for obtaining the conductance, are performed using a density matrix numerical renormalization group (DM-NRG) procedure[1] implemented in the Budapest Flexible DM-NRG code[2].

We keep 3000 states at every iteration with discretization constant $\Lambda = 2$. Conductance is then calculated as

$$G = \frac{e^2}{h} \Gamma \sum_{\sigma, v} \int d\omega \pi A_{\sigma,v}(\omega) \left(-\frac{\partial f(\omega)}{\partial \omega} \right) \tag{4}$$

where $f(\omega)$ is the Fermi function and $A_{\sigma,v}$ is the spectral function associated with level $\{\sigma, v\}$. The differential conductance is then approximated using the equilibrium spectral

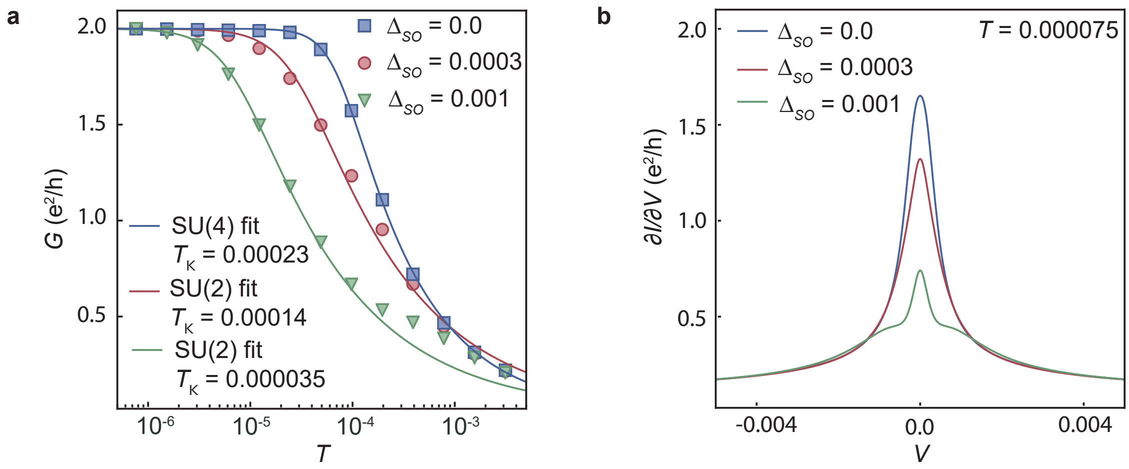

Supplementary Figure 1. SU(4) to SU(2) Kondo effect for $N = 1\,\text{h}$. **a**, Conductance as a function of temperature shows universal scaling laws in the Kondo regime for various SO interactions strengths ($\Delta_{SO} = 0, 0.0003, 0.001$ for SU(4) - blue, SU(2) - red and SU(2) - green, scaling properties). A system with a Δ_{SO} that is slightly larger than T_k can already appear to scale closer to SU(2) universal scaling (red points) **b**, Differential Conductance as a function of bias voltage, calculated using the approximation Supplementary Eq. (5) shows that splitting only occurs for larger SO couplings. Even parameters shown in (a) to exhibit SU(2) scaling (red curve) can still have a single zero-bias peak without side peaks.

function

$$\frac{dI}{dV} = \frac{e^2}{h}\frac{\Gamma}{2}\sum_{\sigma,v}\int d\omega \pi A_{\sigma,v}(\omega)\frac{\partial\left[-f(\omega+V/2)-f(\omega-V/2)\right]}{\partial\omega} \tag{5}$$

Different Kondo models are distinguished by their temperature scaling. As is customary in the literature[3–5], one fits the NRG data to Eq. (1), and extract the parameters s and n. Each model is characterized by different set of (s,n), so comparing the experimental data with Eq. (1), with the different possible values of s and n (Supplementary Fig. 3), one can identify the underlying scenario.

For single occupation $N = 1$ (Supplementary Fig. 1) we use $\Gamma = 0.04$ and $\epsilon_d = -0.4$, where the Coulomb repulsion is taken infinite, and calculate conductance and differential conductance for three values of spin-orbit gap $\Delta_{so} = 0, 0.0003, 0.001$. The red curve in Supplementary Fig. 1a,b agrees with the experimental situation, where the degeneracy is lifted such that scaling follows an SU(2) Kondo scaling while, at the same time, the splitting of the zero-bias peak is not yet resolved.

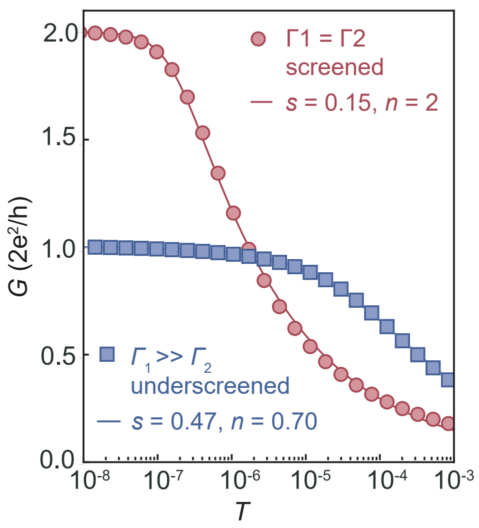

Supplementary Figure 2. Fitting parameters for $N = 2$ h. Conductance as a function of temperature in the spin-triplet grounds state regime for various two scenarios: fully screened (red) and underscreened (blue). A purely emirical scaling law (Eq. (1)) was used without constraint of n and s.

For double occupation $N = 2$ (Supplementary Fig. 3) we use a Coulomb repulsion of $U = 1$ and the level resides at $\epsilon = -1.5$. We use a ferromagnetic interaction $J = -0.1$ to force the system into the spin triplet state forming an $S = 1$ effective model. Then, two scenarios are investigated: (a) The underscreened scenario (blue points in Supplementary Fig. 3), where just one channel participates in the screening $\Gamma_k = 0.1, \Gamma_{k'} = 0.01$. The k' channel Kondo temperature is below the lowest temperature depicted in the plot. (b) The fully screened scenario (red points in Supplementary Fig. 3), where both channels participate equally in the screening $\Gamma_k = \Gamma_{k'} = 0.04$. This is characterized by a different scaling behavior than the underscreened case. The two scenarios are fitted with the empirical curve given by Eq. (1) in the main text. This empirical form was also studied for various $S > 1/2$ Kondo models for the underscreened case[5] with an additional fitting parameter that will not be utilized here, so the parameters we get might deviate slightly from that result. The screened case, which is expected to scale according to Fermi liquid quadratic temperature dependence with scaling parameter $n = 2$, was studied for S=1 with similar results[6]. The empricial fitting parameters are given in the legend to Supplementary Fig. 3. These numerical fits were used in Fig. 3 in the main text to fit the experimental data.

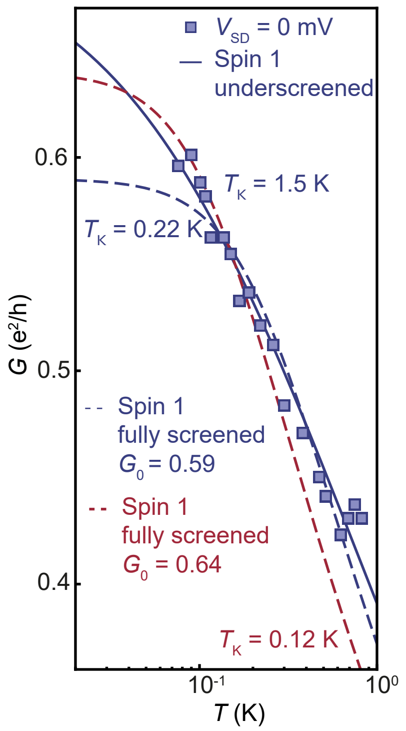

Supplementary Figure 3. Fits for $N = 2\,\mathrm{h}$ with fully screened and underscreened Kondo models. Red and blue dashed lines are fits to the date with the fully screened Kondo model with $G_0 = 0.64$ and $G_0 = 0.59$, respectively. Showing that the underscreened Kondo model (solid blue line) is in better agreement with the data points.

SUPPLEMENTARY REFERENCES

[1]Tóth, A., Moca, C., Legeza, Ö. & Zaránd, G. Density matrix numerical renormalization group for non-abelian symmetries. *Physical Review B* **78**, 245109 (2008).

[2]Legeza, O., Moca, C., Tóth, A., Weymann, I. & Zaránd, G. Manual for the flexible dm-nrg code. *arXiv preprint arXiv:0809.3143* (2008).

[3]Goldhaber-Gordon, D. *et al.* Kondo effect in a single-electron transistor. *Nature* **391**, 156–159 (1998).

[4]Keller, A. J. *et al.* Emergent su (4) kondo physics in a spin–charge-entangled double quantum dot. *Nature Physics* **10**, 145–150 (2014).

[5]Parks, J. *et al.* Mechanical control of spin states in spin-1 molecules and the underscreened kondo effect. *Science* **328**, 1370–1373 (2010).

[6]Blesio, G. G., Manuel, L. O., Aligia, A. A. & Roura-Bas, P. Fully compensated kondo effect

for a two-channel spin s= 1 impurity. *Physical Review B* **100**, 075434 (2019).
